# Supplementary material for: Hierarchical patient-centric caregiver network method for clinical outcomes study
Source: PLoS One. 2019 Feb 13;14(2):e0211218. doi: 10.1371/journal.pone.0211218 (PMC6373908; doi:10.1371/journal.pone.0211218)
Supplement: S1 File — (PDF) [file pone.0211218.s001.pdf]

Hierarchical Patient-centric Caregiver Network Method for Clinical Outcomes Study

Yoonyoung Park*
Panagiotis D. Karampourniotis
Issa Sylla
Amar K. Das

IBM Research, Cambridge, MA

*Yoonyoung.park@ibm.com

S1 Fig A. Degree distributions of caregiver networks
S1 Table A. Results from multivariate regression models predicting length of stay and in-hospital mortality
S1 Table B. Sensitivity Analysis Results

S1 Fig A. Degree distributions of caregiver networks

In a network of caregivers, degree is the number of edges adjacent to each caregiver node.

a) Distribution of degree of caregiver nodes in all-caregiver network, regardless of patient disease type. Average degree was 645.3 with a standard deviation of 453.7.

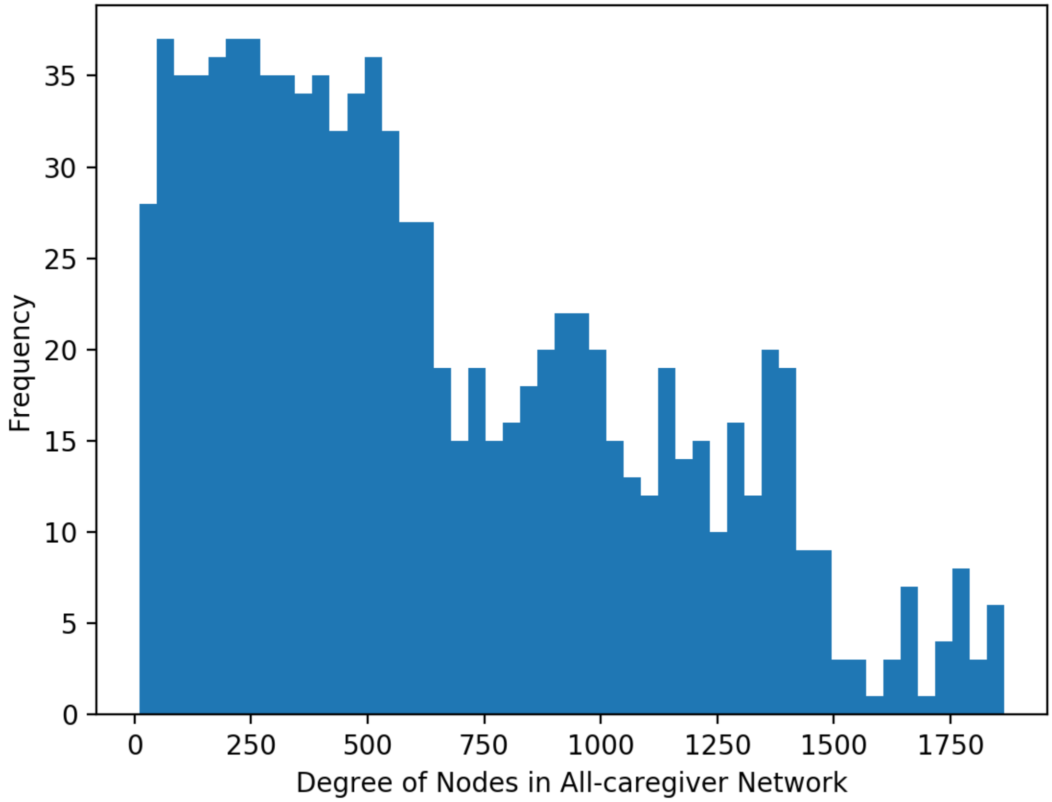

b) Distribution of degree of caregiver nodes in disease-specific caregiver network for patients with coronary heart disease or valve disease. Average degree was 354.1 with a standard deviation of 255.7.

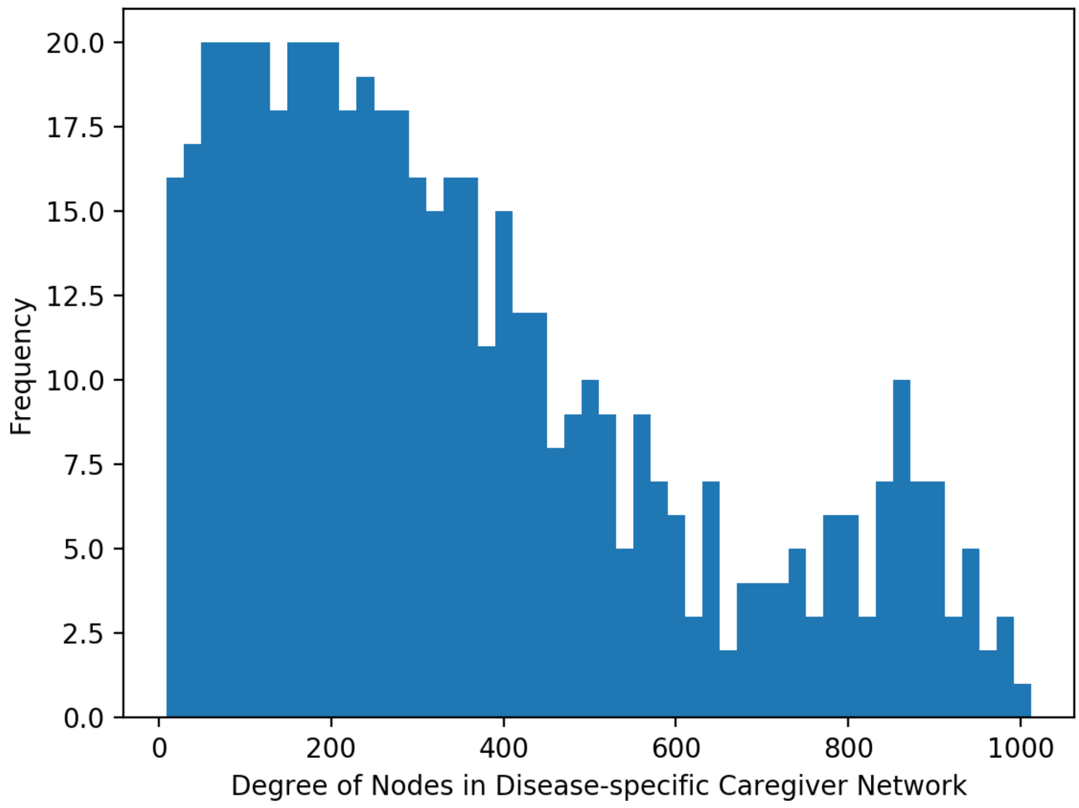

c) Distribution of subnetwork size, i.e. the number of different caregivers that provided care to a patient during a hospitalization period. The average size of a subnetwork was 14.4, meaning that patients encountered 14 to 15 different caregivers on average while they were hospitalized.

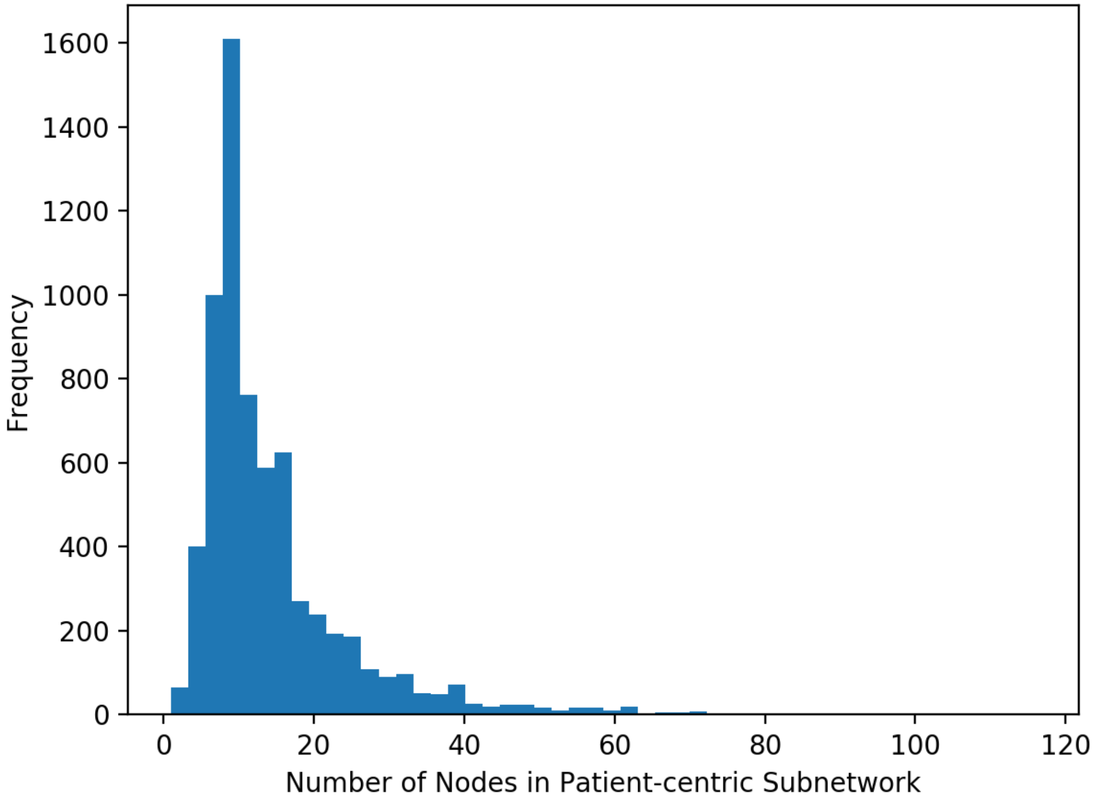

S1 Table A. Results from multivariate regression models predicting length of stay and in-hospital mortality

	Disease-specific				All-caregiver			
	LOS		Death		LOS		Death	
	Coef.	p >|t|	Coef.	p >|t|	Coef.	p >|t|	Coef.	p >|t|
Age	3E-04	0.02	-3E-04	0.79	4E-04	0.01	1E-04	0.91
Female	0.01	0.48	-0.07	0.62	0.01	0.26	-0.07	0.62
Admission[a] - Referral	-0.14	< 0.01	-0.22	0.23	-0.15	< 0.01	-0.15	0.42
Admission - Transfer	-0.06	< 0.01	-0.30	0.06	-0.07	< 0.01	-0.26	0.10
Insurance[b]- Government	0.07	0.03	< 0.01	1.00	0.07	0.02	0.07	0.89
Insurance - Medicaid	0.08	< 0.01	0.41	0.14	0.08	< 0.01	0.34	0.24
Insurance - Private	-0.07	< 0.01	-0.05	0.76	-0.07	< 0.01	-0.05	0.77
Insurance - Self Pay	0.04	0.46	1.43	0.01	0.04	0.52	1.23	0.02
Race/Ethnicity[c] - Asian	-0.06	0.14	-0.19	0.72	-0.06	0.16	-0.21	0.71
Race/Ethnicity - African American	0.02	0.50	-0.23	0.45	0.02	0.54	-0.18	0.55
Race/Ethnicity - Hispanic/Latino	-0.05	0.13	-0.95	0.10	-0.03	0.30	-0.79	0.17
Race/Ethnicity - Others	-0.05	< 0.01	0.49	< 0.01	-0.05	< 0.01	0.54	< 0.01
DRG[d] - Acute CAD	-0.30	< 0.01	3.40	< 0.01	-0.31	< 0.01	2.74	< 0.01
DRG - Other procedures	-0.10	< 0.01	3.04	< 0.01	-0.13	< 0.01	2.54	< 0.01
DRG - PCI	-0.45	< 0.01	1.95	< 0.01	-0.47	< 0.01	1.39	< 0.01
DRG - Valve procedures	0.12	< 0.01	1.34	< 0.01	0.14	< 0.01	1.32	< 0.01
Comorbidity Score	0.09	< 0.01	0.19	< 0.01	0.10	< 0.01	0.19	< 0.01
SAPS	0.05	< 0.01	0.94	< 0.01	0.05	< 0.01	0.99	< 0.01
Mechanical Ventilation	0.08	< 0.01	-0.21	0.29	0.04	0.01	-0.25	0.20
Renal Replacement Therapy	0.09	0.01	0.19	0.50	0.09	0.01	0.24	0.40
DNR or DNI order	0.09	0.01	2.20	< 0.01	0.08	0.02	2.16	< 0.01
Number of Vasopressor Use	0.01	< 0.01	0.20	< 0.01	0.01	< 0.01	0.18	< 0.01
Average betweenness centrality	0.10	< 0.01	-0.12	0.33	0.01	0.20	0.14	0.06
Average degree centrality	-0.18	< 0.01	0.56	0.06	-0.11	< 0.01	1.66	< 0.01
Average clustering coefficients	-0.06	< 0.01	0.22	0.45	-0.11	< 0.01	1.36	< 0.01
Average node experience	-0.03	< 0.01	0.05	0.67	-0.10	< 0.01	0.41	< 0.01
Modularity	0.19	< 0.01	0.02	0.68	0.22	< 0.01	0.13	0.01

[a] Compared to Emergency Admission; [b] Compared to Medicare; [c] Compared to White; [d] Compared to CABG

LOS: length of stay; CAD: coronary artery disease; PCI: percutaneous coronary intervention; CABG: coronary artery bypass graft; SAPS: Simplified Acute Physiology Score; DNR/DNI: Do Not Resuscitate/Do Not Intubate

S1 Table B. Sensitivity Analysis Results

Sensitivity analyses were conducted based on disease-specific caregiver network to examine the robustness of study results. Results presented below are from multivariable regression models predicting either length of stay or in-hospital death.

a) Combining discharge to hospice and in-hospital death as an alternative mortality outcome variable

	LOS		Death	
	Coef.	p >|t|	Coef.	p >|t|
Age	3E-04	0.02	-7E-04	0.60
Female	0.01	0.53	-0.03	0.80
Admission[a] - Referral	-0.14	< 0.01	-0.16	0.36
Admission - Transfer	-0.06	< 0.01	-0.25	0.11
Insurance[b] - Government	0.07	0.03	0.35	0.44
Insurance - Medicaid	0.08	< 0.01	0.39	0.15
Insurance - Private	-0.07	< 0.01	-0.05	0.75
Insurance - Self Pay	0.04	0.46	1.41	0.01
Race/Ethnicity[c] - Asian	-0.05	0.18	-0.04	0.94
Race/Ethnicity - African American	0.02	0.51	-0.26	0.38
Race/Ethnicity - Hispanic/Latino	-0.05	0.13	-0.76	0.14
Race/Ethnicity - Others	-0.05	< 0.01	0.49	< 0.01
DRG[d] - Acute CAD	-0.30	< 0.01	3.35	< 0.01
DRG - Other procedures	-0.10	< 0.01	2.96	< 0.01
DRG - PCI	-0.45	< 0.01	1.80	< 0.01
DRG - Valve procedures	0.12	< 0.01	1.29	< 0.01
Comorbidity Score	0.09	< 0.01	0.21	< 0.01
SAPS	0.05	< 0.01	0.91	< 0.01
Mechanical Ventilation	0.08	< 0.01	-0.30	0.12
Renal Replacement Therapy	0.08	0.02	0.20	0.48
DNR or DNI order	0.11	< 0.01	2.34	< 0.01
Number of Vasopressor Use	0.01	< 0.01	0.18	< 0.01
Average betweenness centrality	0.10	< 0.01	-0.10	0.42
Average degree centrality	-0.19	< 0.01	0.40	0.16
Average clustering coefficients	-0.06	< 0.01	0.08	0.77
Average node experience	-0.03	< 0.01	0.02	0.87
Modularity	0.19	< 0.01	0.04	0.54

[a] Compared to Emergency Admission; [b] Compared to Medicare; [c] Compared to White; [d] Compared to CABG

LOS: length of stay; CAD: coronary artery disease; PCI: percutaneous coronary intervention; CABG: coronary artery bypass graft; SAPS: Simplified Acute Physiology Score; DNR/DNI: Do Not Resuscitate/Do Not Intubate

b) Randomly selecting one admission from each patient (n=6,368)

	LOS		Death	
	Coef.	p >|t|	Coef.	p >|t|
Age	3E-04	0.02	-6E-04	0.66
Female	0.01	0.55	-0.08	0.54
Admission[a] - Referral	-0.14	< 0.01	-0.21	0.25
Admission - Transfer	-0.06	< 0.01	-0.28	0.08
Insurance[b] - Government	0.05	0.14	0.04	0.94
Insurance - Medicaid	0.07	< 0.01	0.44	0.11
Insurance - Private	-0.08	< 0.01	-0.11	0.53
Insurance - Self Pay	0.03	0.62	1.42	0.01
Race/Ethnicity[c] - Asian	-0.06	0.16	-0.39	0.50
Race/Ethnicity - African American	2.2E-03	0.93	-0.29	0.35
Race/Ethnicity - Hispanic/Latino	-0.03	0.43	-0.77	0.17
Race/Ethnicity - Others	-0.05	< 0.01	0.46	< 0.01
DRG[d] - Acute CAD	-0.30	< 0.01	3.41	< 0.01
DRG - Other procedures	-0.11	< 0.01	3.05	< 0.01
DRG - PCI	-0.46	< 0.01	1.92	< 0.01
DRG - Valve procedures	0.12	< 0.01	1.31	< 0.01
Comorbidity Score	0.09	< 0.01	0.19	< 0.01
SAPS	0.06	< 0.01	0.96	< 0.01
Mechanical Ventilation	0.07	< 0.01	-0.27	0.17
Renal Replacement Therapy	0.10	0.01	0.32	0.28
DNR or DNI order	0.10	0.01	2.22	< 0.01
Number of Vasopressor Use	0.01	< 0.01	0.19	< 0.01
Average betweenness centrality	0.10	< 0.01	-0.15	0.24
Average degree centrality	-0.18	< 0.01	0.63	0.03
Average clustering coefficients	-0.06	< 0.01	0.29	0.32
Average node experience	-0.03	< 0.01	0.05	0.69
Modularity	0.19	< 0.01	0.05	0.43

[a] Compared to Emergency Admission; [b] Compared to Medicare; [c] Compared to White; [d] Compared to CABG

LOS: length of stay; CAD: coronary artery disease; PCI: percutaneous coronary intervention; CABG: coronary artery bypass graft; SAPS: Simplified Acute Physiology Score; DNR/DNI: Do Not Resuscitate/Do Not Intubate

c) Using 29* Elixhauser comorbidity variables instead of single comorbidity score value variable

	LOS		Death	
	Coef.	p >|t|	Coef.	p >|t|
Age	3E-04	0.05	3E-04	0.83
Female	0.01	0.37	-0.06	0.66
Admission[a] - Referral	-0.14	< 0.01	-0.22	0.23
Admission - Transfer	-0.06	< 0.01	-0.28	0.09
Insurance[b] - Government	0.06	0.04	-0.35	0.56
Insurance - Medicaid	0.09	< 0.01	0.04	0.89
Insurance - Private	-0.05	< 0.01	-0.22	0.22
Insurance - Self Pay	0.03	0.57	1.05	0.07
Race/Ethnicity[c] - Asian	-0.04	0.26	-0.01	0.98
Race/Ethnicity - African American	0.01	0.66	-0.22	0.48
Race/Ethnicity - Hispanic/Latino	-0.04	0.17	-0.88	0.13
Race/Ethnicity - Others	-0.04	< 0.01	0.42	0.01
DRG[d] - Acute CAD	-0.32	< 0.01	3.50	< 0.01
DRG - Other procedures	-0.11	< 0.01	3.05	< 0.01
DRG - PCI	-0.45	< 0.01	2.05	< 0.01
DRG - Valve procedures	0.08	< 0.01	1.79	< 0.01
SAPS	0.04	< 0.01	0.96	< 0.01
Mechanical Ventilation	0.08	< 0.01	-0.34	0.10
Renal Replacement Therapy	0.03	0.47	0.45	0.17
DNR or DNI order	0.08	0.02	2.35	< 0.01
Number of Vasopressor Use	0.01	< 0.01	0.20	< 0.01
Average betweenness centrality	0.11	< 0.01	-0.14	0.29
Average degree centrality	-0.18	< 0.01	0.62	0.04
Average clustering coefficients	-0.05	0.01	0.25	0.39
Average node experience	-0.02	0.02	0.09	0.43
Modularity	0.18	< 0.01	-1.8E-03	0.98
Congestive heart failure	0.11	< 0.01	-0.14	0.42
Cardiac arrhythmias	0.09	< 0.01	0.01	0.93
Valvular disease	0.04	0.02	-0.80	< 0.01
Pulmonary circulation	0.08	< 0.01	-0.06	0.80
Peripheral vascular	0.06	< 0.01	0.07	0.66
Hypertension	0.04	0.09	-0.28	0.30
Paralysis	0.17	< 0.01	-0.35	0.50
Other neurological disease	0.12	< 0.01	0.92	< 0.01
Chronic pulmonary	0.04	< 0.01	-0.02	0.89

Diabetes uncomplicated	0.01	0.26	0.09	0.55
Diabetes complicated	0.11	< 0.01	0.01	0.96
Hypothyroidism	-0.06	0.35	-0.38	0.72
Renal failure	0.06	0.08	0.14	0.72
Liver disease	0.05	0.32	1.20	0.02
Peptic ulcer disease	-0.09	0.68	-19.80	1.00
AIDS	-3.1E-16	< 0.01	1.4E-07	1.00
Lymphoma	0.06	0.35	-21.37	1.00
Metastatic cancer	0.22	< 0.01	0.63	0.16
Solid tumor	0.09	0.02	-0.17	0.76
Rheumatoid arthritis	0.06	0.54	0.33	0.73
Coagulopathy	0.06	< 0.01	0.83	< 0.01
Obesity	-4E-03	0.85	0.85	< 0.01
Weight loss	0.41	< 0.01	-0.49	0.39
Fluid electrolyte	0.07	< 0.01	0.74	< 0.01
Blood loss anemia	1.0E-18	0.93	1.7E-08	1.00
Deficiency anemias	0.07	< 0.01	-0.98	< 0.01
Alcohol abuse	-0.01	0.71	0.44	0.23
Drug abuse	0.18	< 0.01	-0.66	0.34
Psychoses	0.03	0.40	-0.18	0.73

*There are 30 Elixhauser variables, but depression was not included because there was no patient with depression in the study data.

[a] Compared to Emergency Admission; [b] Compared to Medicare; [c] Compared to White; [d] Compared to CABG

LOS: length of stay; CAD: coronary artery disease; PCI: percutaneous coronary intervention; CABG: coronary artery bypass graft; SAPS: Simplified Acute Physiology Score; DNR/DNI: Do Not Resuscitate/Do Not Intubate; AIDS: acquired immunodeficiency syndrome
